# The Role of Spermidine in Postharvest Fruit Physiology: Effects on Quality Characteristics and Metabolite Content of Sweet Cherry Fruit during Cold Storage

**DOI:** 10.1021/acsomega.4c11222

**Published:** 2025-03-04

**Authors:** Ayşen Melda Çolak, Sadiye Peral Eyduran, Akgul Tas, Oktay Altun, Muttalip Gundogdu, Burhan Ozturk

**Affiliations:** 1 Department of Horticulture, Faculty of Agriculture, Uşak University, Uşak 64000, Turkey; 2 Department of Horticulture, Fethiye Faculty of Agriculture, Muğla Sıtkı Kocman University, Muğla 48000, Turkey; 3 Department of Plant and Animal Production, Seben İzzet Baysal Vocational School, Bolu Abant Izzet Baysal University, Seben Bolu 14750, Turkey; 4 Republic of Türkiye Ministry of Agriculture and Forestry, Erzincan Horticultural Research Institute, Erzincan 24000, Turkey; 5 Department of Horticulture, Faculty of Agriculture, Bolu Abant Izzet Baysal University, Bolu 14030, Turkey; 6 Department of Horticulture, Faculty of Agriculture, Ordu University, Ordu 52000, Turkey

## Abstract

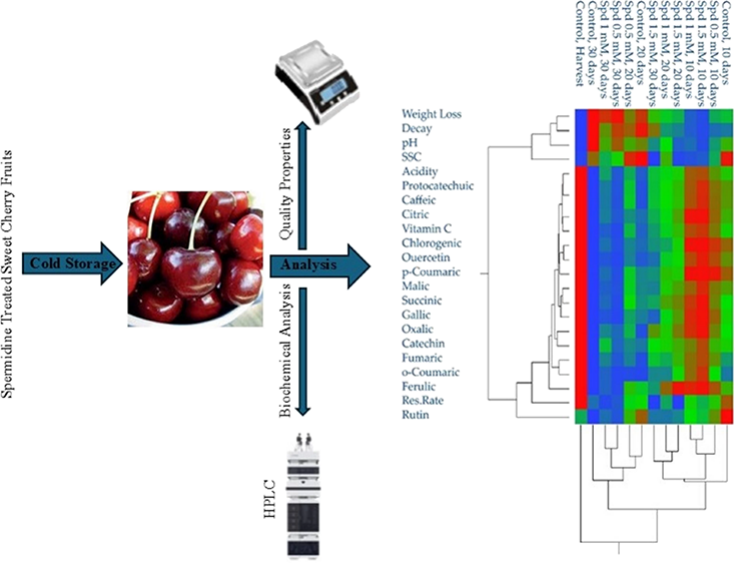

In this study, 0.5, 1.0, and 1.5 mM spermidine were applied to sweet cherry fruit before storage and the fruit were stored for 30 days. In addition, the fruit were stored for 30 days as a control without any treatment. Weight loss, decay rate, soluble solids content (SSC), titratable acidity (TA), pH, respiration rate, phenolic compounds, organic acids, and vitamin C parameters were assessed in fruit during storage. As a result of the study, according to different spermidine treatments, increases in weight loss, SSC, pH, respiration, and decay rates and decreases in titratable acidity were detected in control fruit at different storage periods (10, 20, and 30 days). Among the different spermidine doses applied, it was determined that the highest dose of spermidine applications was 1.5 mM, which prevented the degradation of phenolic compounds, organic acids, and vitamin C contents in sweet cherry fruit. Succinic acid was the dominant organic acid in sweet cherry fruit, with the highest value recorded at 10.30 g kg^–1^ during the harvest period and the lowest value measured at 6.89 g kg^–1^ in the control group at the end of storage (30 days). After succinic acid, malic and citric acids were found to have the highest concentrations, both of which showed a decrease during storage. It was determined that the 1.5 mM dose was the most effective in preventing this decrease. It was also found that gallic acid (30 days: 68.36 mg 100 g^–1^) was the most abundant phenolic compound in the fruit, followed by quercetin (30 days: 9.81 mg 100 g^–1^) and rutin (30 days: 10.09 mg 100 g^–1^), respectively. As a result, it was concluded that exogenous application of spermidine can be used as a postharvest tool in preserving the quality and storage life of sweet cherry fruit.

## Introduction

Sweet cherry (*Prunus avium* L.), a member of the Rosaceae family, is a perennial, fleshy, stone-fruit species.^1^ Its origins are traced to the region encompassing the South Caucasus, the Caspian Sea, and Northeastern Anatolia. Over time, sweet cherry has spread from these gene centers to many parts of the world^2^ and is now cultivated globally, particularly in temperate climates such as the Mediterranean, Central Europe, the USA, Asia, and North Africa.^3^ Sweet cherry trees typically thrive at altitudes of around 1000 m, growing to an average height of 4–5 m, and prefer sandy-clay soils.^1^ Flowering occurs in late March to early April, with flowers approximately 2–2.5 cm in diameter arranged actinomorphically on the branches. The leaves are oval-pointed, are semibright green in spring, and turn orange in autumn, with a glabrous-matte texture.^4^ In Turkey, sweet cherries are primarily produced in provinces such as İzmir, Konya, Manisa, Afyon, and Denizli. They are highly favored by consumers for their vibrant red flesh and pleasant aroma.^5^ Sweet cherry fruit are rich in various biochemicals, including flavonoids, antioxidants, phenolic acids, organic acids, and vitamins.^6,7^ These fruits are known to offer health benefits, such as aiding in the management of diseases like diabetes, cardiovascular issues, inflammation, and particularly cancer.^7,8^ However, factors such as short harvest and storage periods, prolonged handling and transportation, adverse climatic conditions during cultivation, and inadequate preharvest practices like irrigation and fertilization can negatively impact the quality of sweet cherries.^9^ Due to their delicate and perishable nature, maintaining freshness is essential to preserving the beneficial properties of sweet cherry fruit. The limited harvest and storage period of sweet cherries is a key challenge in maintaining fruit freshness. Therefore, there is a pressing need for methods to increase the storage of sweet cherries. In recent years, putrescine and spermidine applications have been widely used to extend the storage period of various perishable fruits and to preserve fruit quality characteristics.^10−12^ Spermidine, one of these compounds called polyamines, is naturally found in all organs and cells of plants and plays an important role in fruit growth, development, and physiology, especially in postharvest life. This compound also has stress-neutralizing effects on plants thanks to its antioxidant properties.^13^ In addition, it is known that quality parameters such as fruit weight, decay and respiration rate, water-soluble solids content, and titratable acidity can be improved with the postharvest application of this compound in fruits such as nectarines and strawberries.^14,15^

This study aimed to investigate the effects of 0.5, 1.0, and 1.5 mM spermidine treatments on various physical and biochemical properties of sweet cherry fruit stored for different periods (10, 20, and 30 days). There is currently no direct research on impact of spermidine applications on the phytochemical properties of stored sweet cherry fruit,^14−16^ and it is anticipated that this study will contribute to advancing research in this area.

## Materials and Methods

### Materials

Sweet cherry fruit (cv. '0900 Ziraat') patterns were hand-harvested from in Yalova province, chosen based on specific cultivar criteria. After harvesting, the samples were placed in labeled containers, stored in ice boxes, and promptly transported to laboratory for analysis. The experimental setup involved three replicates (1 kg of fruit per replicate), three spermidine treatments (0.5, 1.0, and 1.5 mM), and storage periods of 10, 20, and 30 days (d). Accordingly, the study took into account the work of Orman^14^ in the selection of specific spermidine doses. The fruits were immersed in spermidine solutions of 0.0 mM (control, pure water) and 0.5 1.0 mM, and 1.5 mM for 30 min. Following the treatment, the fruit were dried with blotting paper, packaged in perforated polyethylene (PE) bags (30 cm × 40 cm, 0.01 mm thickness, 1.0 kg capacity), and stored under cold conditions (0 ± 0.5 °C and 90 ± 5% relative humidity) for up to 30 days. Measurements were performed at harvest (beginning of storage) and every 10 days throughout the storage period for all treatments, including the control.

### Identification of Weight Loss and Decay Rate

To assess the weight loss of sweet cherry fruit during storage, a digital precision balance with a sensitivity of 0.01 g was used. The weight loss was figured outed as a percentage by employing formula provided by Hosseini et al.:^9^
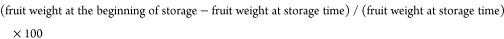


Here, the initial fruit weight refers to the fresh weight of the cherries before storage, while the fruit weight at storage time corresponds to the weight of the same cherries measured at a given point during storage. This formula enables researchers to quantify the percentage reduction in fruit weight over time, reflecting moisture loss, or other factors contributing to weight changes.

The decay rate of the cherries was evaluated using a subjective scoring system to categorize the extent of decay observed on the fruit surface. The decay scoring scale was defined as follows: 0, no visible decay on the fruit; 1, slight decay, affecting no more than 25% of the fruit’s surface; 2, middle decay, affecting between 25 and 50% of the fruit’s surface; 3, severe decay, with more than 50% of the fruit’s surface affected. To quantify the overall decay rate of cherries, the method defined by Cao et al.^17^ was applied. The decay rate was calculated by the following formula:
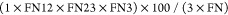
where FN is the total number of fruit, and FN1, FN2 and FN3 are fruit numbers representing different decay scores.

### Identification of Soluble Solids Content, Titratable Acidity, pH, and Respiration Rate

The soluble solids content (SSC) was measured with a portable hand-held refractometer (ATC, BX50, Turkey). Titratable acidity (TA) was specified by titrating 10 mL of juice diluted with 10 mL of distilled water against 0.1 N NaOH, using phenolphthalein as a marker, as per Hanif et al.^18^ pH was measured using a benchtop pH meter (Thermo, Orion Star A111, USA). To determine the respiration rate, 100 g of fruit per replicate was placed in 2000 mL glass bottles, with a CO_2_ sensor probe (Testo 535, Germany) inserted through a hole in the bottle cap. The probe and cap were sealed with parafilm to hamper CO_2_ escape.^19^ All experiments were conducted in a controlled environment; the respiration rate was recorded in mg CO_2_ kg^–1^ h^–1^. Data were determined by the following formulas.
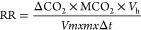
ΔCO_2_ = CO_2_(*t*_2_) *–* CO_2_(*t*_1_)
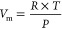
where

RR: respiration rate, mg CO_2_ kg^–1^ h^–1^

ΔCO_2_: CO_2_ volumetric concentration, ppm, 10^–6^ L L^–1^

MCO_2_: CO_2_ gas molecular weight, 44.01 g mol^–1^

*V*_h_: bottle volume, L.

*m*: weight of single kernel wheat with shells, 1 kg

Δ*t*: trial period, h.

CO_2_(*t*1): initial CO_2_ concentration, ppm, 10^–6^ L L^–1^

CO_2_(*t*2): CO_2_ concentration at the end of the trial period, ppm, 10^–6^ L L^–1^

*V*_m_: molar volume of gas, L mol^–1^

*R*: gas constant: 0.08206 L^–1^ mol^–1^ 1 K^–1^

*T*: temperature, K

*P*: pressure, atm.

### Identification of Organic Acids

Organic acids were quantified using calibration curves prepared with standards (100–800 ppm, 99% purity, Sigma-Aldrich, Taufkirchen, Germany). A 250 g portion of sweet cherry was homogenized, and 50 g of the homogenate was diluted with distilled water (1:3). An extraction solvent of 25 mL of 0.009 N H_2_SO_4_ was added, and the mixture was shaken for 1 h and then centrifuged at 15.000 × *g* for 15 min. The supernatant was filtered through coarse paper and a 0.45 μm membrane (Millipore, Germany) and purified with a Sep-Pak C18 cartridge. Analysis was performed using an Agilent 1100 HPLC system with a Bio-Rad HPX-87H column. The mobile phase was 0.009 N H_2_SO_4_, with detection at 254 and 280 nm.^20^ Results were expressed as milligrams per 100 g of FW based on calibration curves.

### Vitamin C Determination

Vitamin C in sweet cherry extracts (100 g) was extracted using 2.5% (w/v) metaphosphoric acid (Sigma, M6285, 33.5%) and centrifuged at 6500 rpm for 10 min at 4 °C. 0.5 mL was diluted to 10 mL with the same acid solution. The supernatant was filtered through a 0.45 μm PTFE syringe filter (Phenomenex, UK) and analyzed on a C18 column (Phenomenex Luna C18, 250 × 4.60 mm, 5 μ) at 25 °C. The mobile phase was acidified by double-distilled water (pH 2.2, H_2_SO_4_) at a flow rate of 1 mL/min. Detection was performed at 254 nm by using a DAD detector. Quantification was based on l-ascorbic acid standards (Sigma A5960) at 50, 100, 500, 1000, and 2000 ppm,^21^ with results expressed as mg/100 g FW.

### Identification of Phenolic Compounds

Phenolic compounds were analyzed using a modified HPLC method.^22^ Standards for gallic acid, protocatechuic acid (20–120 ppm), chlorogenic acid (200–1200 ppm), and caffeic, vanillic, ferulic, and *p*-coumaric acids (50–300 ppm) were prepared along with calibration curves (Sigma-Aldrich, 99% purity, Taufkirchen, Germany). Flavonoid standards included rutin (50–300 ppm), quercetin (100–600 ppm), and catechin (500–2500 ppm). Fruit samples (100 g) were homogenized, shaken (Heidolph Unimax 1010, Germany) for 1 h, and diluted with water (1:1). The extract was centrifuged (15.000 rpm, 15 min), filtered (coarse paper and 0.45 μm membrane), and analyzed via HPLC (Agilent, USA) using a 250 × 4.6 mm, 4 μm, ODS column (HiChrom, USA). Mobile phases were methanol:acetic acid:water (10:2:28 and 90:2:8). Detection was at 254 and 280 nm, with a flow rate of 1 mL/min and an injection volume of 20 μL. Results were expressed as milligrams per 100 g of fresh weight.

### Statistical Analysis

The characteristics of sweet cherry fruit were analyzed using two-way ANOVA to evaluate the effects of two independent factors and their interaction. Posthoc comparisons were performed with Student’s *t* test, with significance set at *P* < 0.05. Statistical analyses were conducted using JMP 13 software (SAS Institute Inc., Cary, NC, USA), enabling detailed examination of main and interaction effects. Results included *F*-values, *P*-values, and treatment group means with standard errors to ensure clarity and transparency. This approach ensured robust and reproducible conclusions about factors influencing sweet cherry quality.

## Results and Discussion

### Weight Loss, SSC, Titratable Acidity, and pH

**Figure 1:**
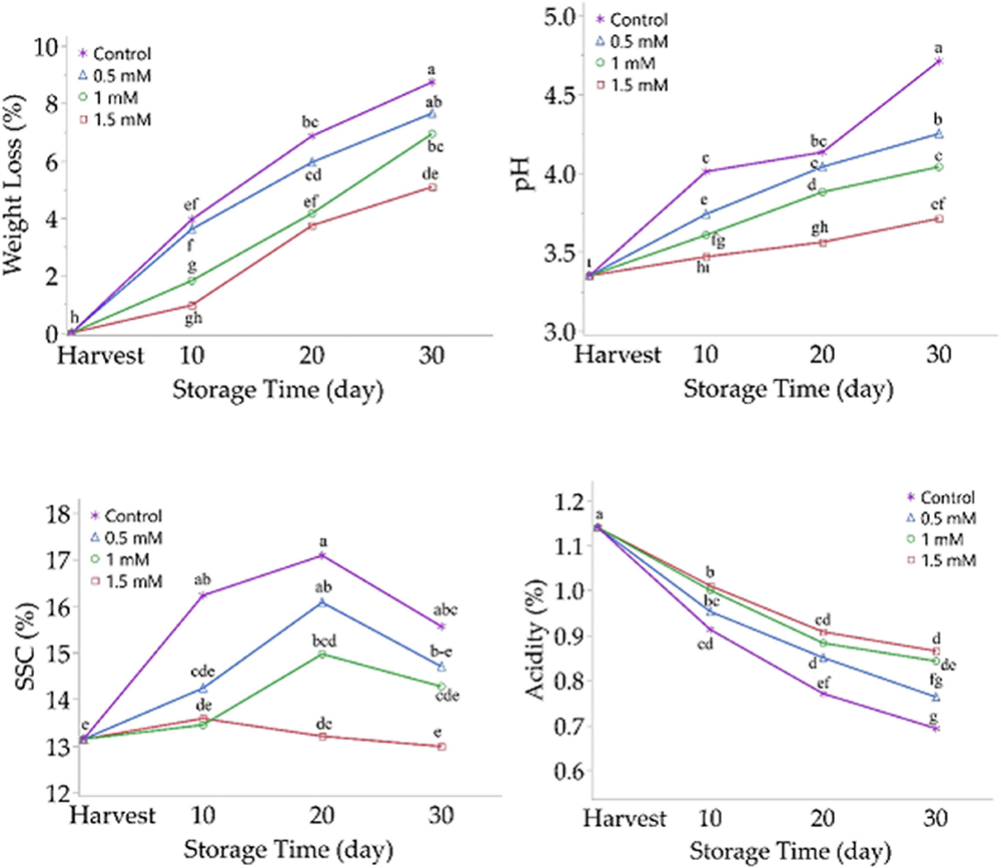
Effect of spermidine application on the quality properties of sweet cherry fruit. Different letters on top of the storage periods indicate significant differences at *p* ≤ 0.05.

The differences between the weight losses of control and spermidine applications during storage of cherry fruit were significant (*P* < 0.001). In control and all treatment dose spermidine applied fruit, weight losses also increased as storage period increased (10, 20, and 30 days). When all spermidine treatments were compared with control, it was determined that weight losses could change according to different storage periods. In the study, in all three storage periods, significantly less weight losses were observed in spermidine treatments with different treatment doses compared to control fruit. Hence, after 10 days, fruit treated with 0.5 mM spermidine dose (3.61%), 1.0 mM spermidine dose (1.82%), and 1.5 mM spermidine dose (0.96%) had considerably less weight losses than the control (3.95%). Similarly, after 30 days in study, significantly less weight loss was observed in fruit with 0.5 mM spermidine dose (7.65%), 1.0 mM spermidine dose (6.93%), and 1.5 mM spermidine dose (5.08%) compared to control (8.73%) fruit. In addition, among the different doses of spermidine used in the study at three different storage periods, the least weight loss was observed in fruit with a 1.5 mM spermidine dose (Table 1 and Figure 1). In this study, there are literature research examples related to various fruits that support the results determined regarding weight loss in sweet cherry. Orman^14^ found that, after 30 days of storage, significantly less weight loss was observed in nectarine fruit of “Fantasia” variety to which different doses of spermidine were applied, in fruit applied with 1.5 mM spermidine. Jalali et al.^15^ reported that after 12 days of storage, weight loss was minimal in strawberry fruit to which they applied 1.0 and 1.5 mM spermidine. Gündoğdu et al.^16^ reported that after 20 and 40 days of cold storage, weight loss was prevented more in fruit applied with 1.5 mM spermidine, compared to control fruit. Physiological weight loss is generally due to various metabolic activities, such as respiration and transpiration. In this study, spermidine applications with different treatment doses significantly reduced the weight loss of sweet cherry fruit in all three storage periods. This may be due to the lower respiration rate in spermidine-applied fruit (Table 1). Mirdehghan et al.^23^ reported that applying polyamines like spermidine to various stored fruits enhanced membrane fluidity, maintained membrane integrity, reduced water loss, and thereby minimized weight loss. Similarly, Zahedi et al.^24^ observed that spermidine application increased the antioxidant activity of fruit tissues in stored fruits, which helped prevent oxidative damage to the plasma membrane and further reduced fruit weight loss. The differences between the SSC values of different spermidine applications and the control during storage of cherry fruit were significant (*P* < 0.001). In the study, in three storage periods (10, 20, and 30 days), dramatically low SSC amounts were observed in spermidine treatments with different treatment doses than in control fruit. Accordingly, after 10 days, significantly low SSC amounts were detected in fruit treated with 0.5 mM spermidine dose (14.24%), 1.0 mM spermidine dose (13.45%), and 1.5 mM spermidine dose (13.59%) than in control fruit. Similarly, after 30 days in study, considerably low SSC amounts were detected in fruit treated with 0.5 mM spermidine dose (14.70%), 1.0 mM spermidine dose (14.28%), and 1.5 mM spermidine dose (12.99%) than control fruit (15.57%). In the study, the lowest SSC amounts were observed in fruit with 1.0 mM spermidine after 10 days and in fruit with 1.5 mM spermidine after 20 and 30 days (Table 1). Orman^14^ reported that in nectarine fruit treated with different doses of spermidine, significantly low SSC amounts were observed in fruit treated with both 1.0 and 1.5 mM spermidine at the end of 30 days of storage, compared to control fruit. Jalali et al.^15^ reported that in strawberry fruit to which different doses of spermidine were applied, lowest SSC amounts were observed in fruit with 1.5 mM spermidine after 12 days of storage. In this study, when the results obtained regarding SSC in cherries were compared with results of the literature studies regarding some of the fruit mentioned above, it was observed that certain spermidine applications significantly reduced SSC amounts in fruit. Accordingly, the reason why some spermidine dose treatments had lower SSC amounts compared to the control application at different storage times in this study and in the literature examples may be related to the respiration rates detected at lower rates compared to control in this study (Table 1). This is because it is known that there is a direct proportion between the reducing effect of spermidine applications on SSC amounts in some fruit and the decrease in respiration rates and ethylene production of treated fruit.^25^

**Table 1:** Effect of Different Spermidine Dose Treatments on the Quality Characteristics of Sweet Cherry Fruit at Different Storage Periods^a^

**storage time**		**weight loss (%)**	**decay rate (%)**	**SSC (%)**	**acidity (%)**	**pH**	**respiration rate(mg CO**_**2**_kg^–1^ h^–1^**)**
harvest		0.00 ± 0.00d	0.00 ± 0.00c	13.15 ± 0.25a	1.14 ± 0.04a	3.35 ± 0.02c	22.72 ± 1.18a
10th day		2.59 ± 0.36c	1.96 ± 0.23c	14.38 ± 0.53a	0.97 ± 0.01b	3.71 ± 0.06bc	11.47 ± 0.69b
20th day		5.18 ± 0.39b	5.68 ± 0.69b	15.34 ± 0.43a	0.85 ± 0.02c	3.90 ± 0.06b	8.37 ± 0.55c
30th day		7.10 ± 0.45a	8.09 ± 0.50a	14.38 ± 0.26a	0.79 ± 0.02c	4.18 ± 0.10a	6.28 ± 0.25d
storage time × spermidine							
harvest		0.00 ± 0.00h	0.00 ± 0.00h	13.15 ± 0.25e	1.14 ± 0.04a	3.35 ± 0.02i	22.72 ± 1.18a
10th day	control	3.95 ± 0.36ef	2.74 ± 0.25ef	16.23 ± 0.92ab	0.91 ± 0.02cd	4.01 ± 0.03c	15.55 ± 0.89b
	spd 0.5 mM	3.61 ± 0.60f	2.36 ± 0.55ef	14.24 ± 1.76cde	0.95 ± 0.02bc	3.74 ± 0.07e	10.99 ± 0.61c
	spd 1 mM	1.82 ± 0.27g	1.86 ± 0.15fg	13.45 ± 0.24de	1.00 ± 0.01b	3.61 ± 0.04fg	10.39 ± 0.29cd
	spd 1.5 mM	0.96 ± 0.11gh	0.89 ± 0.09gh	13.59 ± 0.06de	1.01 ± 0.02b	3.47 ± 0.04hi	8.94 ± 0.21de
20th day	control	6.86 ± 0.29bc	8.80 ± 0.72b	17.09 ± 0.60a	0.77 ± 0.01ef	4.13 ± 0.03bc	10.17 ± 0.56cde
	spd 0.5 mM	5.94 ± 0.59cd	7.41 ± 0.23cd	16.08 ± 0.66ab	0.85 ± 0.07d	4.04 ± 0.03c	9.57 ± 0.48cde
	spd 1 mM	4.16 ± 0.34ef	3.49 ± 0.77e	14.97 ± 0.18bcd	0.88 ± 0.02cd	3.88 ± 0.07d	8.70 ± 0.46ef
	spd 1.5 mM	3.74 ± 0.53ef	3.02 ± 0.27ef	13.21 ± 0.18de	0.91 ± 0.01cd	3.56 ± 0.04gh	5.04 ± 0.14i
	control	8.73 ± 0.13a	10.90 ± 0.28a	15.57 ± 0.05abc	0.69 ± 0.02g	4.71 ± 0.03a	7.38 ± 0.29fg
	spd 0.5 mM	7.65 ± 0.59ab	8.02 ± 0.46bc	14.70 ± 0.28b–e	0.76 ± 0.01fg	4.25 ± 0.03b	6.75 ± 0.33gh
30th day	spd 1 mM	6.93 ± 0.81bc	6.43 ± 0.71d	14.28 ± 0.05cde	0.84 ± 0.00de	4.04 ± 0.05c	5.79 ± 0.19hi
	spd 1.5 mM	5.08 ± 0.79de	7.03 ± 0.51cd	12.99 ± 0.28e	0.87 ± 0.02d	3.71 ± 0.04ef	5.19 ± 0.18i
ANOVA							
*F*_Storagetime_		35.32***	35.07***	2.34^ns^	33.85***	12.83***	69.47***
*F*_storagetime×spermidine_		29.68***	56.32***	4.54***	20.13***	71.09***	81.28***

a Different letters in the same column indicate statistical differences at *p* ≤ 0.05 level. *** indicates *p* ≤ 0.001 level; ns: not significant.

During storage of sweet cherry fruit, the differences between TA values of control and different spermidine treatments were significant (*P* < 0.001). In the study, at three storage periods (10, 20, and 30 days), significantly higher TA values were observed in spermidine treatments with different treatment doses than in control fruit. Thus, after 10 days, significantly higher TA values were detected in fruit treated with 0.5 mM spermidine dose (0.95%), 1.0 mM spermidine dose (1.00%), and 1.5 mM spermidine dose (1.01%) than in control fruit. After 20 days, significantly higher TA values were observed in fruit treated with 0.5 mM spermidine dose (0.85%), 1.0 mM spermidine dose (0.88%), and 1.5 mM spermidine dose (0.91%) than the control (0.77%). Similarly, after 30 days in study, significantly higher TA values were detected in fruit treated with 0.5 mM spermidine dose (0.76%), 1.0 mM spermidine dose (% 0.84), and 1.5 mM spermidine dose (0.87%) than the control fruit (0.69%). In addition, among the different doses of spermidine used in the study, the highest TA values were observed in fruit with 1.5 mM spermidine in three different storage periods (Table 1). Some researchers reported that polyamine applications increased fruit titratable acid content in various fruit stored for certain periods.^25,26^ Based on this, Davarynejad et al.^25^ stated that polyamine application in stored fruit significantly reduced the respiration rate and ethylene biosynthesis processes, thus preserving the fruit TA better. In the study, the differences between the pH values of spermidine fruit and control fruit are important (*P* < 0.001). In all three storage periods, significantly lower pH values were observed in different treatment doses of spermidine applications than in control fruit. Accordingly, after 10 days, significantly lower pH values were detected in fruit treated with 0.5 mM spermidine dose (3.74), 1.0 mM spermidine dose (3.61), and 1.5 mM spermidine dose (3.47) than in control fruit (4.01). After 20 days, significantly lower pH values were observed in fruit treated with 0.5 mM spermidine dose (4.04), 1.0 mM spermidine dose (3.88), and 1.5 mM spermidine dose (3.56) than the control (4.13). Similarly, after 30 days, significantly lower pH values were observed in fruit treated with 0.5 mM spermidine dose (4.25), 1.0 mM spermidine dose (4.04), and 1.5 mM spermidine dose (3.71) than control fruit (4.71). In addition, among the different spermidine dose applications used in the study, the lowest pH values were observed in fruit with 1.5 mM spermidine dose at all three storage periods (Table 1). Similar to these results observed in sweet cherries, Orman^14^ observed significantly lower pH values in nectarine fruit to which 0.5 1.0 and 1.5 mM spermidine was applied, compared to control fruit, in all fruit to which spermidine doses were applied after 30 days of storage. Accordingly, regarding the findings reached in the studies, Davarynejad et al.^25^ stated that polyamine application in stored fruit preserved the TA of the fruit more by reducing the respiration rate and ethylene synthesis; thus, pH values, which have an inverse correlation relationship with TA, decreased.

### Decay Rate and Respiration Rate

**Figure 2:**
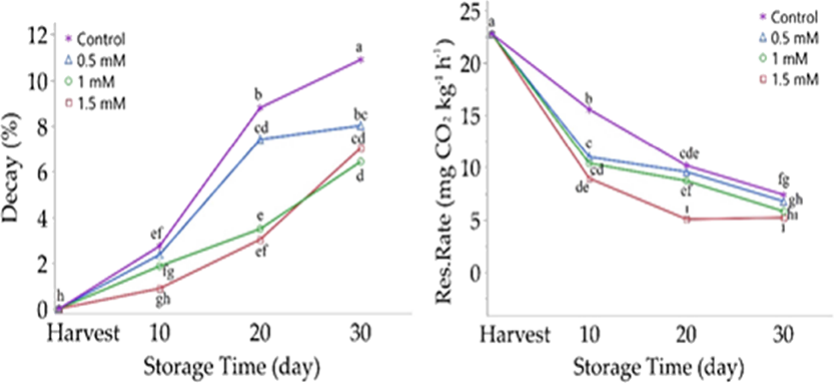
Effect of spermidine application on the decay and respiration rate of sweet cherry fruit. Different letters on top of the storage periods indicate significant differences at *p* ≤ 0.05.

During storage of sweet cherry fruit (10, 20, and 30 days), the differences between the decay rates of different spermidine treatments and the control were significant (*P* < 0.001). Hence, after 10 days, significantly low decay rates were detected in fruit with 1.0 mM spermidine dose (1.86%) and 1.5 mM spermidine dose (0.89%) than in control fruit (2.74%); similar decay rates were observed between fruit treated with 0.5 mM spermidine dose (2.36%) and control fruit (2.74%). In addition, after 20 days, significantly low decay rates were found in fruit with 0.5 mM spermidine dose (7.41%), 1.0 mM spermidine dose (3.49%), and 1.5 mM spermidine dose (3.02%) than the control (8.80%). Similarly, after 30 days in study, significantly low decay rates were found in fruit with 0.5 mM spermidine dose (8.02%), 1.0 mM spermidine dose (6.43%), and 1.5 mM spermidine dose (7.03%) than the control (10.90%). In addition, the lowest decay rates were observed in fruit with 1.5 mM spermidine after 10 and 20 days and in fruit with 1.0 mM spermidine after 30 days (Table 1 and Figure 2).

Consistent with the findings on decay rates in sweet cherries from this study, Orman^14^ reported that nectarine fruits treated with different doses of spermidine exhibited significantly less decay after 30 days, with the 1.0 mM spermidine treatment showing the greatest reduction compared to the control group. Jalali et al.^15^ reported that in strawberry fruit to which different doses of spermidine were applied, lowest decay rates were observed in fruit to which 1.5 mM spermidine was applied, after 6 and 12 days of storage. Accordingly, regarding findings obtained in studies on decay, Davarynejad et al.^25^ reported that application of polyamine to stored fruit slows down the rate of fruit ripening by reducing the respiration rate and ethylene synthesis, and thus, decay activity can be significantly delayed in the fruit with a slower ripening rate. The differences between the control application and different spermidine applications in terms of respiration rates (mg CO_2_ kg^–1^ h^–1^) are significant (*P* < 0.001). Thereby, after 10 days, significantly lower respiration rates were found in fruit with 0.5 mM spermidine dose (10.99), 1.0 mM spermidine dose (10.39), and 1.5 mM spermidine dose (8.94) than in control (15.55). After 20 days, significantly lower respiration rates were detected in fruit with 1.0 mM spermidine dose (8.70) and 1.5 mM spermidine dose (5.04) than in control fruit (10.17), while similar respiration rates were observed between fruit treated with 0.5 mM spermidine dose (9.57) and control fruit (10.17). On the other hand, after 30 days, significantly lower respiration rates were determined in fruit treated with 0.5 mM spermidine dose (6.75), 1.0 mM spermidine dose (5.79), and 1.5 mM spermidine dose (5.19) compared to control (7.38). In addition, among different doses of spermidine used in the study, the lowest respiration rates were observed in fruit with a 1.5 mM spermidine dose at three different storage periods (Table 1). Similar to these findings, Orman^14^ reported that in nectarine fruit with different doses of spermidine, after 30 days, fruits treated with 1.5 mM spermidine had a significantly low respiration rate than control fruit. Gündoğdu et al.^16^ stated that in plum fruit with different doses of spermidine, after 20 and 40 days of cold storage, fruits with 1.5 mM spermidine had a low respiration rate than control fruit. Accordingly, regarding the findings reached in terms of respiration rate in various fruits in the studies, several researchers have stated that there is a direct relationship between slowed respiration rates in polyamine-treated fruit and delay in fruit ripening.^27^ Nonetheless, Hanif et al.^18^ stated that lower CO_2_ amounts were secreted in polyamine-applied fruit, metabolic activity decreased, and respiratory rate decreased due to these effects.

### Phenolic Compounds

During storage of sweet cherry fruit (10, 20, and 30 days), phenolic compounds and their amounts detected in control and fruit treated with different spermidine doses are important (*P* < 0.001). In terms of phenolic compounds and their amounts detected in sweet cherry fruit, only rutin, all phenolic compound amounts, except for rutin, gradually decreased both in control fruit and in different dose-treated fruit as storage periods increased. In the study, except for the amounts of rutin compound detected only in 0.5, 1.0, and 1.5 mM spermidine-treated fruit stored for 10 and 20 days (Table 3), significantly higher phenolic compound amounts were detected in all three spermidine treatments at the end of all storage periods (Tables 2 and 3). In fact, this situation shows that phenolic compounds decrease more slowly in fruit treated with different doses of spermidine compared to control fruit during certain storage periods. This situation has been associated with the prevention of losses of phenolic compounds in fruit by different doses of spermidine applications. In the study, it was determined that among different doses of spermidine applications, 1.5 mM dose spermidine application generally prevented phenolic compound losses more than control fruit. Hence, in all storage periods of sweet cherry fruit, the best result was given by 1.0 mM dose spermidine application, which contains more *p*-coumaric acid, compared to control, in all phenolic compounds detected in fruit [gallic acid, chlorogenic acid, ferulic acid, caffeic acid, *o*-coumaric acid, protocatechuic acid, quercetin, catechin, *p*-coumaric acid (except for the 20-day storage period; the best result was given by 1.0 mM dose spermidine application, which contains more *p*-coumaric acid, compared to control in this storage period), and rutin (except for the 10- and 20-day storage periods; significantly lower levels of rutin were observed in all three spermidine applications compared to control in these storage periods)], and the least loss of phenolic compounds occurred during storage in this treatment dose spermidine application (Tables 2 and 3). It was determined that gallic acid was most abundant phenolic compound in sweet cherry fruit, followed by quercetin and rutin compounds, respectively. Accordingly, after 30 days, gallic acid, quercetin, and rutin contents were determined as 68.36, 9.81, and 10.09 mg 100g^–1^, respectively (Tables 2 and 3). Sweet cherries are particularly rich in phenolic compounds.^28^ The above results regarding phenolic compounds in sweet cherry are parallel to the results of some other literature studies conducted on this subject in various fruits.

**Table 2:** Effect of Different Spermidine Dose Treatments on Phenolic Compounds Detected (mg/100g) in Sweet Cherry Fruit at Different Storage Periods^a^

**storage time**		**caffeic**	**catechin**	**chlorogenic**	**ferulic**	**gallic**
harvest		7.96 ± 0.29a	4.32 ± 0.14a	1.76 ± 0.03a	5.04 ± 0.09a	99.34 ± 1.89a
10th day		6.50 ± 0.38b	3.88 ± 0.12b	1.64 ± 0.05b	4.74 ± 0.15b	88.85 ± 5.21b
20th day		5.07 ± 0.80c	3.66 ± 0.15c	1.36 ± 0.07c	4.61 ± 0.20b	78.77 ± 4.18c
30th day		4.15 ± 0.81d	3.50 ± 0.22d	1.25 ± 0.09d	3.85 ± 0.25c	68.36 ± 2.73d
storage time × spermidine						
harvest		7.96 ± 0.29a	4.32 ± 0.14a	1.76 ± 0.03a	5.04 ± 0.09a	99.34 ± 1.89a
10th day	control	6.04 ± 0.24d	3.76 ± 0.04de	1.57 ± 0.01d	4.54 ± 0.09e	81.23 ± 1.00f
	spd 0.5 mM	6.40 ± 0.21c	3.81 ± 0.02cde	1.62 ± 0.03c	4.72 ± 0.07cd	88.10 ± 1.42d
	spd 1 mM	6.60 ± 0.11bc	3.91 ± 0.05c	1.67 ± 0.01b	4.81 ± 0.09bc	91.69 ± 1.34c
	spd 1.5 mM	6.94 ± 0.18b	4.02 ± 0.11b	1.70 ± 0.02b	4.87 ± 0.05b	94.36 ± 1.17b
20th day	control	3.81 ± 0.19h	3.49 ± 0.08gh	1.28 ± 0.03h	4.36 ± 0.05f	74.15 ± 0.76h
	spd 0.5 mM	5.23 ± 0.28f	3.55 ± 0.06g	1.33 ± 0.02g	4.51 ± 0.07e	76.62 ± 1.09g
	spd 1 mM	5.49 ± 0.23ef	3.76 ± 0.06de	1.37 ± 0.01f	4.69 ± 0.07d	79.59 ± 1.67f
	spd 1.5 mM	5.74 ± 0.23de	3.83 ± 0.01cd	1.46 ± 0.05e	4.86 ± 0.06b	84.72 ± 0.50e
	control	3.16 ± 0.25i	3.19 ± 0.09i	1.14 ± 0.01j	3.56 ± 0.08j	65.13 ± 1.26k
	spd 0.5 mM	3.81 ± 0.13h	3.42 ± 0.04h	1.21 ± 0.02i	3.72 ± 0.07i	67.08 ± 1.00j
30th day	spd 1 mM	4.45 ± 0.13g	3.66 ± 0.03f	1.29 ± 0.02h	3.93 ± 0.06h	69.74 ± 1.00i
	spd 1.5 mM	5.16 ± 0.51f	3.72 ± 0.07ef	1.35 ± 0.02fg	4.20 ± 0.05g	71.49 ± 1.42i
ANOVA						
*F*_storagetime_		52.94***	31.56***	118.14***	73.25***	99.77***
*F*_storagetime×spermidine_		124.12***	65.6***	269.16***	184.02***	303.73***

a Different letters in the same column indicate statistical differences at *p* ≤ 0.05 level. *** indicates *p* ≤ 0.001 level; ns: not significant.

**Table 3:** Continuation of Table 2 (mg/100 g)^a^

**storage time**		***o*-coumaric**	***p*-coumaric**	**protocatechuic**	**quercetin**	**rutin**
harvest		8.93 ± 0.25a	6.35 ± 0.19a	1.37 ± 0.05a	14.55 ± 0.11a	10.67 ± 0.36bc
10th day		6.06 ± 1.08b	6.05 ± 0.17b	1.11 ± 0.06b	12.92 ± 0.43b	11.75 ± 1.17a
20th day		4.71 ± 0.57c	5.32 ± 0.23c	0.87 ± 0.14c	10.89 ± 0.61c	11.06 ± 0.73b
30th day		4.40 ± 0.42c	4.62 ± 0.34d	0.71 ± 0.14d	9.81 ± 0.44d	10.09 ± 0.33c
storage time × spermidine						
harvest		8.93 ± 0.25a	6.35 ± 0.19a	1.37 ± 0.05a	14.55 ± 0.11a	10.67 ± 0.36fg
10th day	control	4.76 ± 0.18e	5.81 ± 0.09d	1.04 ± 0.03de	12.30 ± 0.15d	13.63 ± 0.17a
	spd 0.5 mM	5.39 ± 0.18d	6.02 ± 0.08c	1.09 ± 0.04cd	12.96 ± 0.20c	11.65 ± 0.16c
	spd 1 mM	6.72 ± 0.16c	6.14 ± 0.05bc	1.13 ± 0.03bc	12.97 ± 0.08c	10.95 ± 0.09e
	spd 1.5 mM	7.37 ± 0.23b	6.23 ± 0.05ab	1.19 ± 0.03b	13.43 ± 0.07b	10.78 ± 0.09ef
20th day	control	4.20 ± 0.18g	4.98 ± 0.04g	0.65 ± 0.03i	10.08 ± 0.13i	12.09 ± 0.12b
	spd 0.5 mM	4.28 ± 0.21fg	5.30 ± 0.11f	0.89 ± 0.05g	10.62 ± 0.11g	11.31 ± 0.07d
	spd 1 mM	4.88 ± 0.28e	5.51 ± 0.07e	0.94 ± 0.04fg	11.28 ± 0.16f	10.36 ± 0.13h
	spd 1.5 mM	5.47 ± 0.30d	5.48 ± 0.10e	0.99 ± 0.04ef	11.56 ± 0.11e	10.49 ± 0.17gh
	control	4.00 ± 0.21g	4.18 ± 0.13j	0.54 ± 0.05j	9.29 ± 0.06k	9.65 ± 0.25j
	spd 0.5 mM	4.17 ± 0.39g	4.48 ± 0.12i	0.66 ± 0.03i	9.58 ± 0.09j	10.06 ± 0.20i
30th day	spd 1 mM	4.57 ± 0.12ef	4.78 ± 0.05h	0.77 ± 0.03h	10.00 ± 0.12i	10.30 ± 0.10hi
	spd 1.5 mM	4.88 ± 0.18e	5.03 ± 0.11g	0.88 ± 0.09g	10.38 ± 0.09h	10.36 ± 0.13h
ANOVA						
*F*_storage time_		50.63***	103.68***	52.17***	171.95***	11.79***
*F*_storage time×spermidine_		159.68***	196.42***	117.26***	762.07***	145***

a Different letters in the same column indicate statistical differences at *p* ≤ 0.05 level. *** indicates *p* ≤ 0.001 level; ns: not significant.

Orman^14^ reported that chlorogenic acid, quercetin, and rutin were most dominant phenolic compounds detected in nectarine fruit treated with different doses of spermidine as a result of 30 days of storage and that phenolic compound losses were better prevented in fruit treated with 1.5 mM spermidine among different doses of spermidine. Gündoğdu et al.^16^ reported that chlorogenic acid was the most dominant phenolic compound detected in plum fruit treated with different doses of spermidine as a result of 20 and 40 days of cold storage and that phenolic compound losses were less in fruit treated with 1.5 mM spermidine among different doses of spermidine. In this study and above literature studies supporting this study, it is thought that results obtained in terms of various phenolic compounds may be related to “phenol oxidase enzyme”, which causes internal chemical changes in polyamine-treated fruit to occur more slowly than in control fruit.^29^ In addition, it should be taken into account that amounts of phenolic compounds present in fruits, which can vary seasonally, may vary depending on many factors such as fruit type and variety, growing conditions, climate characteristics, and storage conditions.^30^

### Organic Acids and Vitamin C

During storage of sweet cherry fruit (10, 20, and 30 days), amounts of organic acids and vitamin C detected in fruit with different spermidine dose applications are significant (*P* < 0.001). Thus, after all storage periods in the study, significantly higher organic acid and vitamin C amounts were detected in all three (0.5, 1.0, and 1.5 mM) spermidine applications compared to control fruit (Table 4), and all different dose spermidine applications significantly prevented organic acid and vitamin C losses in fruit. In the study, after a 30-day storage of sweet cherry fruit, it was determined that among different dose spermidine applications, 1.5 mM dose spermidine application generally prevented organic acid and vitamin C losses more than control fruit. In addition, dominant organic acid of sweet cherry fruit was succinic acid, followed by vitamin C and fumaric acid, respectively. Accordingly, after 30 days of storage, succinic acid, vitamin C, and fumaric acid contents were determined as 10.30 g kg^–1^, 10.09 mg 100 g^–1^, and 3.53 mg kg^–1^, respectively (Table 4). The results of this study determined above regarding organic acids and vitamin C in sweet cherry are similar to the results of some other literature studies conducted on this subject in various fruits. Orman^14^ reported that malic acid was the most dominant organic acid among organic acids detected in nectarine fruit applied with different doses of spermidine, as a result of 30 days of storage and that there low less organic acid losses in fruit applied with 1.5 mM spermidine among different doses of spermidine. Gündoğdu et al.^16^ stated that malic acid was most dominant organic acid among organic acids detected in plum fruit to which different doses of spermidine were applied, as a result of 20 and 40 days of cold storage, and that among different doses of spermidine applications, organic acid losses were prevented more in fruit to which 1.5 mM spermidine was applied. Jalali et al.^15^ stated that the amounts of vitamin C were significantly higher in fruit to which 1.5 mM spermidine was applied than in control fruits, as a result of 3 and 6 days of storage periods in strawberry fruit to which 1.0 and 1.5 mM spermidine doses were applied. Mortazavi et al.^31^ applied different spermidine doses to the strawberry fruit named “Selva”, and after 10 days of storage, highest vitamin C amounts were found in fruit with 1.5 mM spermidine. Accordingly, in this study and above literature studies supporting this study, in terms of the results determined regarding vitamin C, some researchers reported that the application of polyamines such as spermidine in stored fruit reduced the ascorbate oxidase enzyme activity in the fruit and thus the vitamin C amounts were better preserved.^32^ However, amounts of organic acids and vitamin C in fruit may vary depending on many factors such as fruit type and variety, storage conditions, and genetic characteristics.^33^

**Table 4:** Effect of Different Spermidine Dose Treatments on Organic Acid (g kg^–1^; Fumaric: mg kg^–1^) and Vitamin C (mg 100 g^–1^) Detected in Sweet Cherry Fruit at Different Storage Periods^a^

**storage time**		**citric**	**malic**	**succinic**	**oxalic**	**fumaric**	**vitamin C**
harvest		2.28 ± 0.17b	2.29 ± 0.40b	9.21 ± 0.26b	1.12 ± 0.10b	3.23 ± 0.14b	8.74 ± 0.43b
10th day		1.83 ± 0.20c	1.68 ± 0.34c	8.48 ± 0.36c	0.93 ± 0.16c	3.06 ± 0.13c	7.74 ± 0.45c
20th day		1.55 ± 0.22d	1.45 ± 0.32c	7.42 ± 0.38d	0.83 ± 0.18d	2.70 ± 0.16d	6.95 ± 0.45d
30th day		2.63 ± 0.11a	3.43 ± 0.14a	10.30 ± 0.07a	1.32 ± 0.05a	3.53 ± 0.02a	10.09 ± 0.15a
Storage time × spermidine							
harvest		2.63 ± 0.11a	3.43 ± 0.14a	10.30 ± 0.07a	1.32 ± 0.05a	3.53 ± 0.02a	10.09 ± 0.15a
10th day	control	2.06 ± 0.07d	1.69 ± 0.08fg	8.90 ± 0.09d	0.99 ± 0.02d	3.04 ± 0.06e	8.09 ± 0.13d
	spd 0.5 mM	2.22 ± 0.06c	2.32 ± 0.13d	9.12 ± 0.07c	1.09 ± 0.04c	3.19 ± 0.05d	8.70 ± 0.15c
	spd 1 mM	2.40 ± 0.01b	2.50 ± 0.15c	9.24 ± 0.05c	1.18 ± 0.01b	3.31 ± 0.04c	9.01 ± 0.04b
	spd 1.5 mM	2.45 ± 0.09b	2.67 ± 0.16b	9.58 ± 0.09b	1.23 ± 0.05b	3.39 ± 0.05b	9.15 ± 0.12b
20th day	control	1.60 ± 0.07h	1.25 ± 0.09h	7.97 ± 0.10g	0.70 ± 0.05h	2.88 ± 0.03f	7.09 ± 0.10g
	spd 0.5 mM	1.73 ± 0.03fg	1.59 ± 0.09g	8.40 ± 0.12f	0.92 ± 0.02e	3.05 ± 0.03e	7.74 ± 0.07ef
	spd 1 mM	1.92 ± 0.04e	1.75 ± 0.06f	8.65 ± 0.05e	1.01 ± 0.05d	3.14 ± 0.03d	7.87 ± 0.09e
	spd 1.5 mM	2.09 ± 0.05d	2.15 ± 0.06e	8.89 ± 0.09d	1.10 ± 0.06c	3.19 ± 0.05d	8.25 ± 0.19d
	control	1.24 ± 0.05j	1.02 ± 0.06i	6.89 ± 0.11j	0.60 ± 0.05i	2.52 ± 0.07i	6.46 ± 0.18i
	spd 0.5 mM	1.49 ± 0.03i	1.34 ± 0.06h	7.34 ± 0.04i	0.78 ± 0.01g	2.62 ± 0.05h	6.70 ± 0.11h
30th day	spd 1 mM	1.66 ± 0.05gh	1.60 ± 0.08g	7.54 ± 0.03h	0.85 ± 0.03f	2.75 ± 0.05g	7.05 ± 0.06g
	spd 1.5 mM	1.81 ± 0.06f	1.83 ± 0.06f	7.90 ± 0.11g	1.07 ± 0.02c	2.92 ± 0.03f	7.58 ± 0.16f
ANOVA							
*F*_storagetime_		57.29***	121.48***	57.29***	19.29***	44.32***	80.46***
*F*_storagetime×spermidine_		184.85***	528.76***	161.31***	117.47***	174.63***	270.84***

a Different letters in the same column indicate statistical differences at *p* ≤ 0.05 level. *** indicates *p* ≤ 0.001 level; ns: not significant.

### Statistical Analysis of Relationships among Quality Characteristics, Organic Acids, Phenolic Compounds, and Effects of Spermidine Treatments

**Figure 3:**
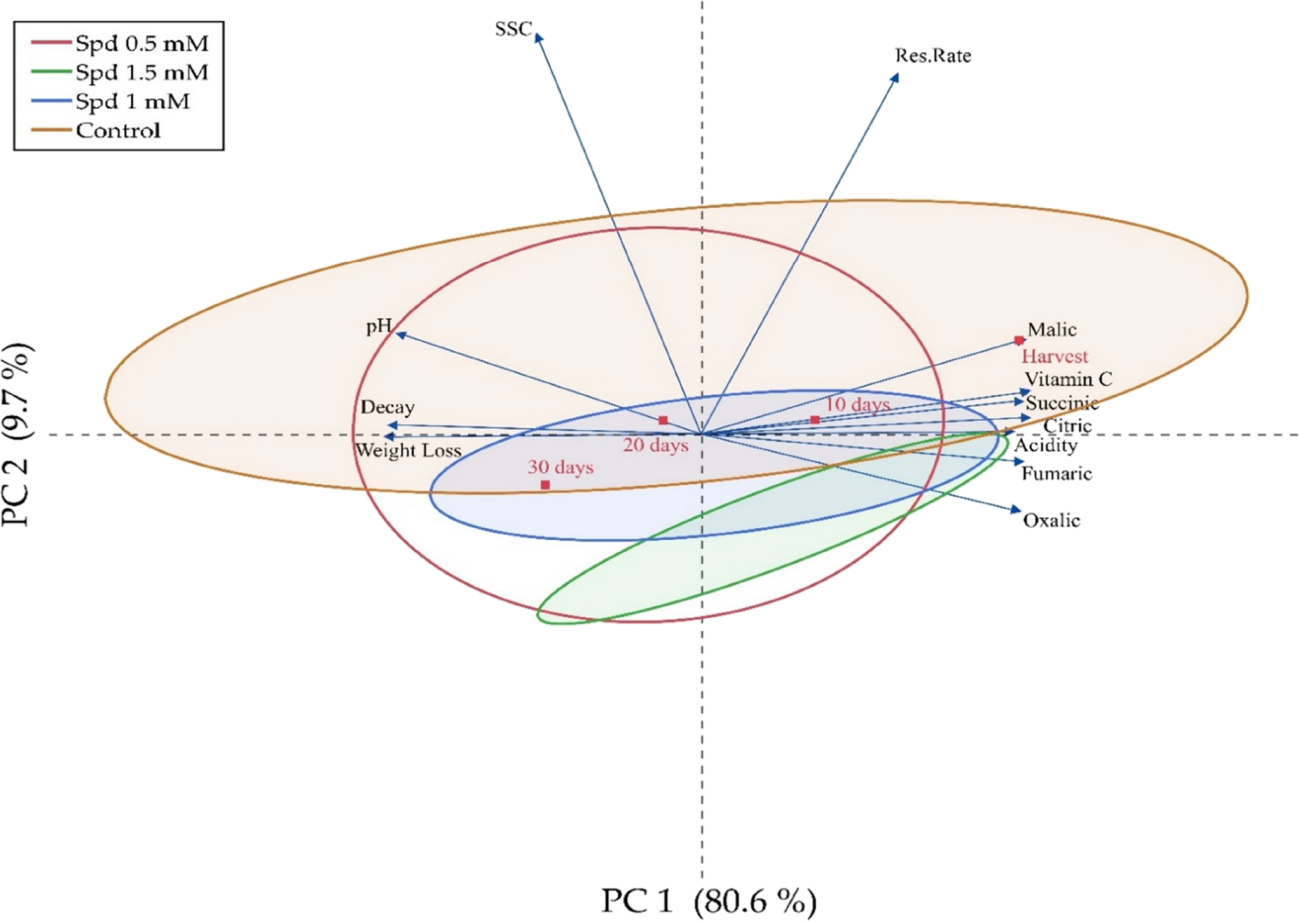
Identification of the relationship between spermidine, organic acid, and quality properties and storage by PCA.

This study evaluated the influence of various spermidine treatments on the quality parameters and biochemical composition of sweet cherry fruit during storage. The investigation employed advanced statistical methods, including principal component analysis (PCA), heatmap visualization, and correlation analysis, to uncover patterns and relationships in the data. PCA was performed to determine the major sources of variance among the measured parameters. PCA indicated that variations in spermidine treatments and organic acids collectively accounted for 90.3% of the total variance in the data set. Organic acid levels were highest at harvest and showed a steady decline during storage. Among the organic acids, succinic acid, vitamin C, and fumaric acid were the most abundant. A positive correlation was observed among the organic acids, suggesting that they exhibited parallel trends during storage. Spermidine treatments had overlapping effects in the PCA plot, with the 30-day storage period showing the most significant changes in organic acid profiles (Figure 3).

**Figure 4:**
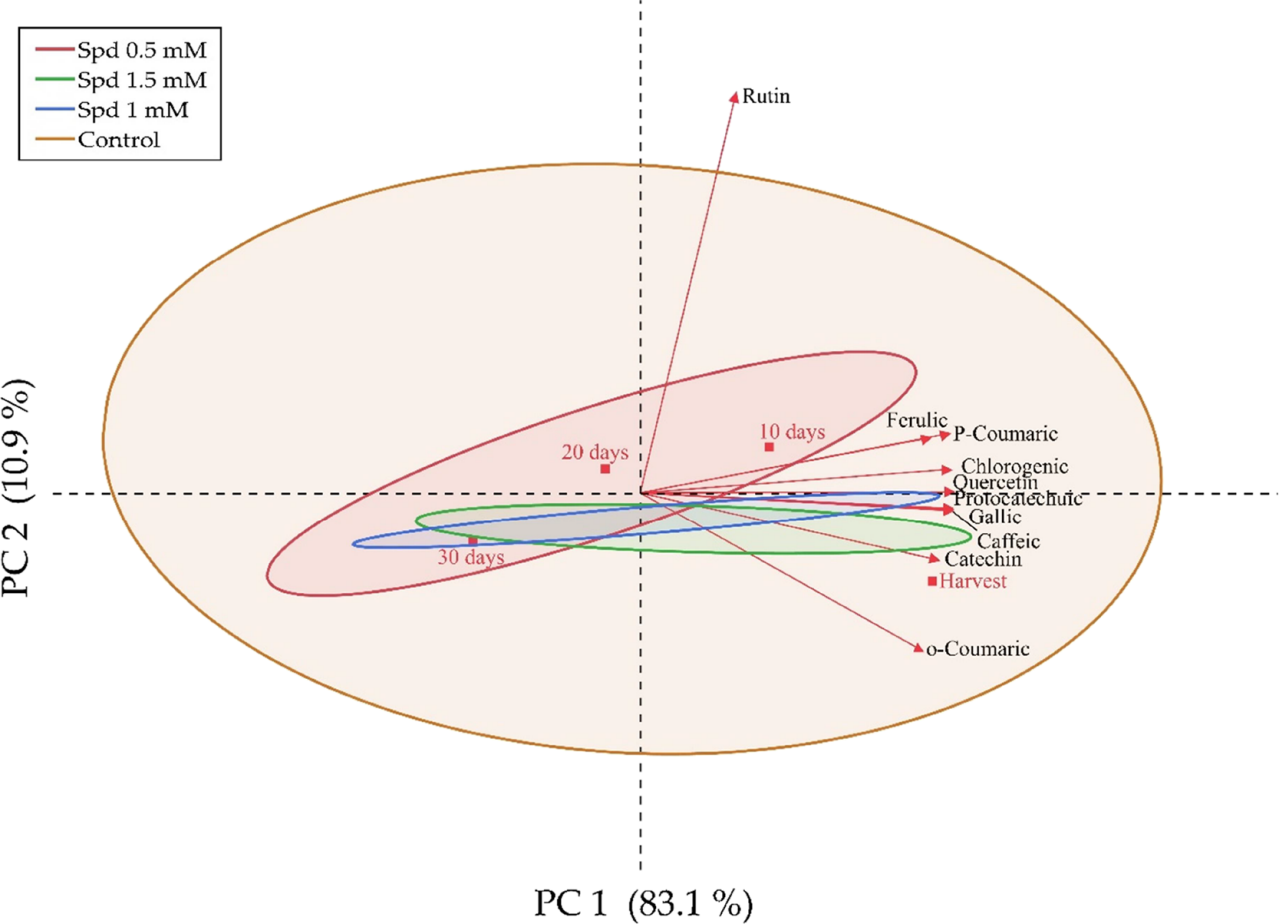
Identification of the relationship between phenolic compounds, spermidine, and storage time by PCA.

The phenolic composition contributed to 94.0% of the total variance in PCA. Specific phenolic compounds demonstrated distinct temporal changes: Catechin and *o*-coumaric acid concentrations decreased significantly by day 10. Conversely, the levels of caffeic acid, gallic acid, and quercetin increased during storage. Rutin emerged as the most concentrated phenolic compound across all treatments (Figure 4).

**Figure 5:**
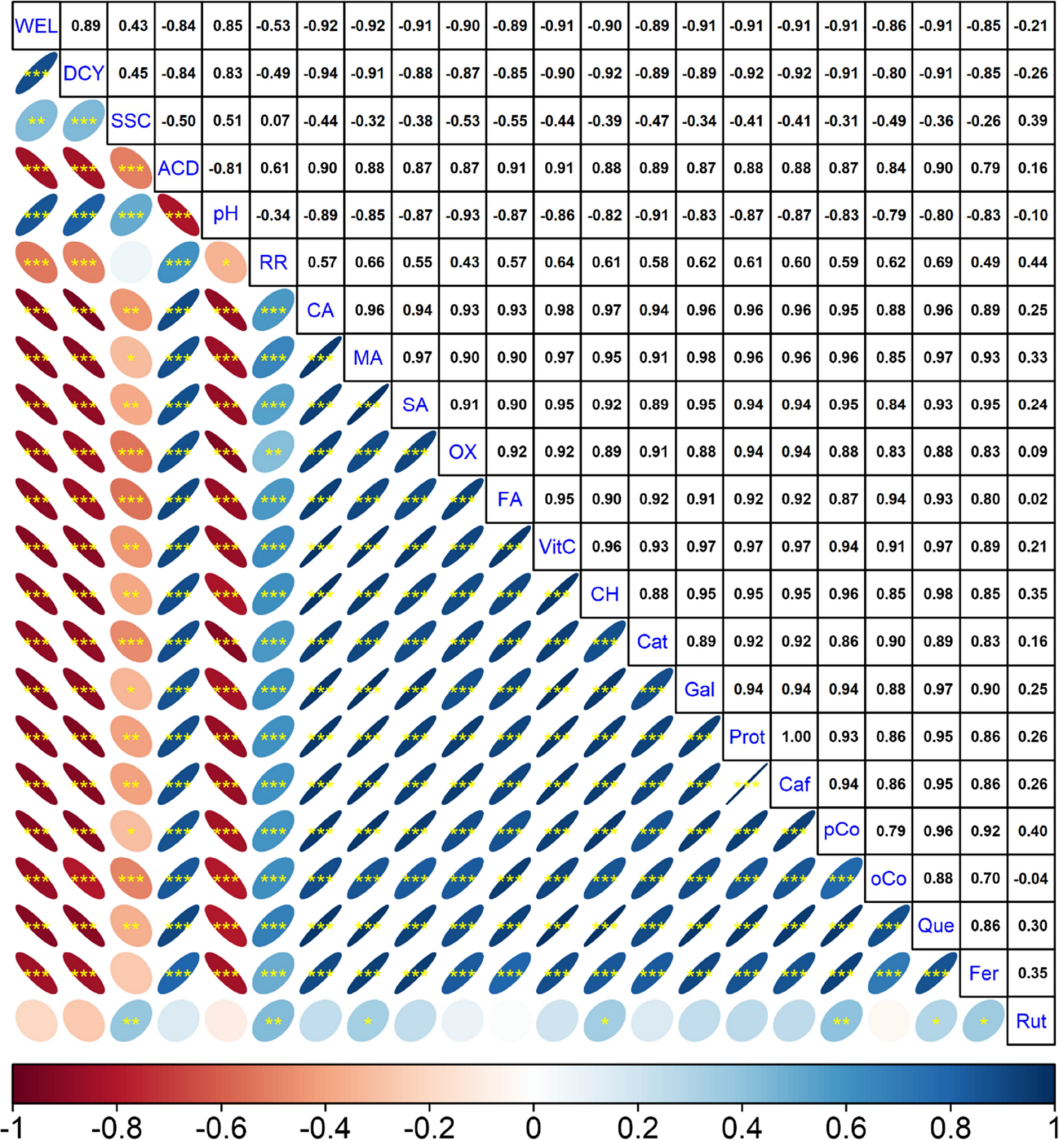
Identification of correlations between quality traits, organic acids, and phenolic compounds. The color scale fading from red to blue indicates correlation values from −1 to +1. *, **, and *** indicate significance at *p* ≤ 0.05, *p* ≤ 0.01, and *p* ≤ 0.001, respectively. WEL: weight loss, DCY: decay, SSC: soluble solid contents, ACD: acidity, RR: respiration rate, CA: citric acid, MA: malic acid, SA: succinic acid, OX: oxalic acid, FU: fumaric acid, VitC: vitamin C, CH: chlorogenic acid, Cat: catechin. Gal: gallic acid, Prot: protocatechuic acid, Caf: caffeic acid, *p*-Co: *p*-coumaric acid, *o*-Co: *o*-coumaric acid, Fer: ferulic acid, Rut: rutin.

Correlation analysis revealed key relationships among quality characteristics, organic acids, and phenolic compounds. Weight loss and decay: A strong positive correlation was observed between weight loss and decay (*r* = 0.89, *p* ≤ 0.001), indicating that fruit with higher weight loss experienced more pronounced decay. Weight loss was also positively correlated with SSC and pH, suggesting that these attributes change concurrently as fruit loses moisture during storage. Vitamin C and organic acids: Vitamin C exhibited strong positive correlations with other organic acids, reflecting their shared metabolic pathways and a simultaneous decline over time. Significant positive correlations were identified among the various phenolic compounds (*p* ≤ 0.001), indicating coordinated fluctuations in their levels during storage (Figure 5).

**Figure 6:**
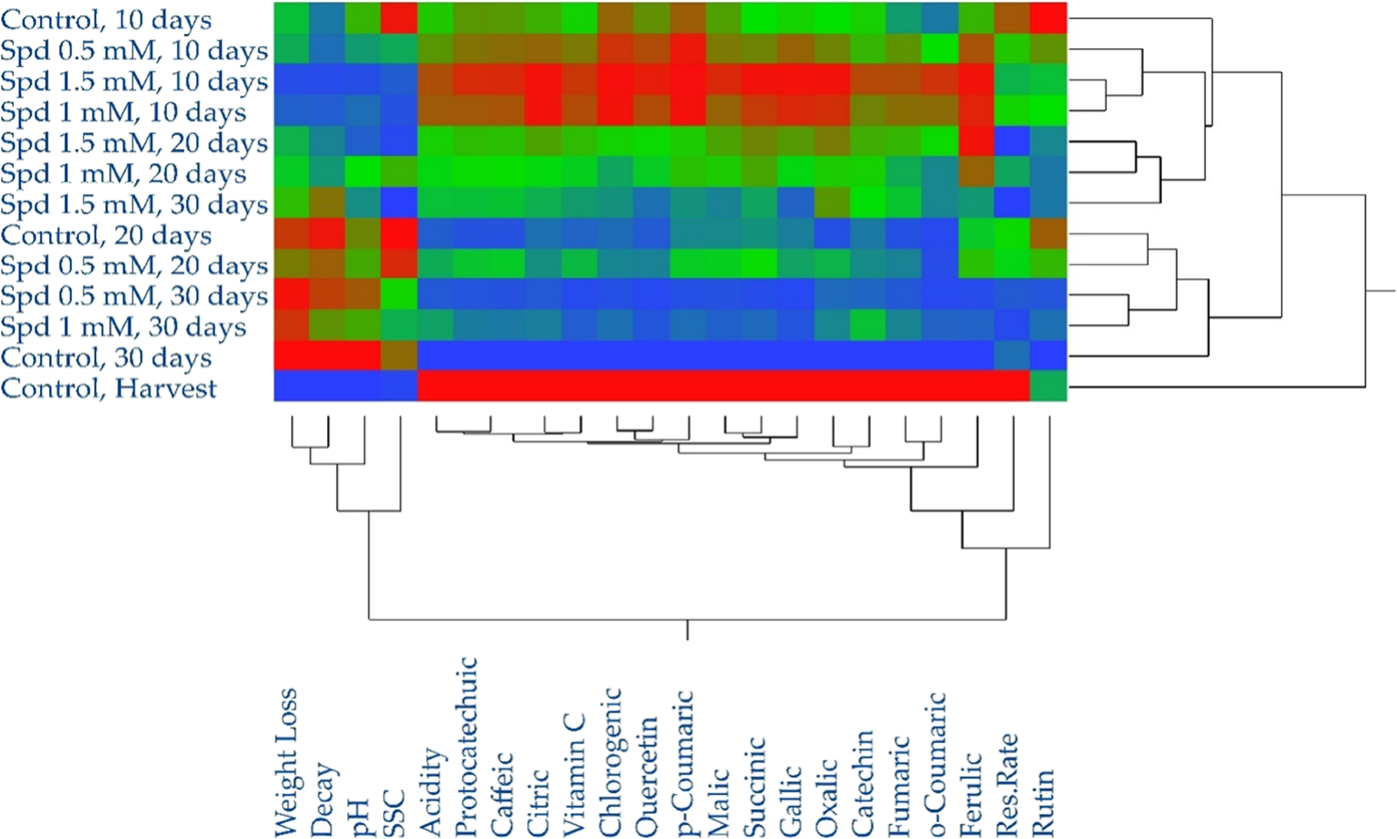
Determination of the relationship between phenolic compounds, spermidine, and storage time by heatmap. From blue to red scale color indicates values from minimum to maximum for each property.

Heatmap analysis provided a visual representation of the biochemical changes during storage. Distinct differences were evident among the storage periods with the harvest period forming a separate cluster from the subsequent storage stages. The highest concentrations of organic acids and phenolic compounds were recorded at harvest and after treatment with 1.5 mM spermidine on day 10. The lowest levels of these compounds were found in control and in 0.5 mM spermidine treatment on day 30, suggesting that higher spermidine concentrations better preserved biochemical quality (Figure 6). Spermidine treatments significantly influenced the quality and biochemical composition of sweet cherries during storage. The 1.5 mM spermidine treatment was particularly effective in maintaining higher levels of organic acids and phenolic compounds. Temporal changes: Storage time had a profound impact, with the greatest biochemical changes observed by day 30. Quality correlations: Strong relationships among quality parameters, organic acids, and phenolic compounds highlight the interconnected nature of these attributes in fruit physiology. Conclusions: The integration of PCA, heatmap visualization, and correlation analysis provided a comprehensive understanding of how spermidine treatments and storage duration influence the sweet cherry quality. The results highlight the potential of spermidine, especially at higher concentrations, to preserve the biochemical and sensory properties of sweet cherries during long-term storage.

## Conclusions

In this study, the effects of spermidine on physical quality parameters and bioactive contents of stored sweet cherries were investigated. Spermidine had positive effects on maintaining fruit quality by significantly reducing the quality losses in fruit. In the study, significantly lower weight loss was observed in fruits treated with spermidine at different doses (0.5, 1.0, and 1.5 mM) across three storage periods (10, 20, and 30 days) compared to the control group. Among the spermidine treatments, the 1.5 mM dose resulted in the least weight loss. In terms of SSC, the least SSC amounts were observed in fruit with 1.0 mM spermidine after 10 days and in fruit with 1.5 mM spermidine after 20 and 30 days. In addition, in terms of TA value, significantly higher TA values were detected in spermidine treatments with different treatment doses in all three storage periods; among the different dose spermidine treatments, the highest TA values were detected in fruit with a 1.5 mM spermidine dose. Also, significantly lower pH values were observed in spermidine treatments with different treatment doses in all three storage periods. In terms of fruit respiration rate, among different doses of spermidine treatments, the lowest respiration rates were detected in fruit with 1.5 mM spermidine dose in all three storage periods. In addition, in terms of fruit decay, the least decay was observed in fruit with 1.5 mM spermidine dose after 10 and 20 days and in fruit with 1.0 mM spermidine dose after 30 days. However, among the different spermidine doses applied, it was determined that the 1.5 mM dose of spermidine application prevented degradation of phenolic compounds, organic acids, and vitamin C contents in sweet cherry fruit. In literature studies, spermidine is recommended to be used in improving postharvest quality parameters of fruits, based on economic benefit criteria. Similarly, as a result of this study, it was concluded that the exogenous application of spermidine can be used as a postharvest tool to protect the quality and storage life of sweet cherry fruit.
